# Supplementary material for: Estimation of spatial demographic maps from polymorphism data using a neural network
Source: bioRxiv. 2024 Mar 17:2024.03.15.585300. Preprint. [Version 1] doi: 10.1101/2024.03.15.585300 (PMC10980082; doi:10.1101/2024.03.15.585300)
Supplement: Supplement 1 [file NIHPP2024.03.15.585300v1-supplement-1.pdf]

bioRxiv preprint doi: https://doi.org/10.1101/2024.03.15.585300; this version posted March 17, 2024. The copyright holder for this preprint (which was not certified by peer review) is the author/funder, who has granted bioRxiv a license to display the preprint in perpetuity. It is made available under aCC-BY 4.0 International license.

Supplementary material

bioRxiv preprint doi: https://doi.org/10.1101/2024.03.15.585300; this version posted March 17, 2024. The copyright holder for this preprint (which was not certified by peer review) is the author/funder, who has granted bioRxiv a license to display the preprint in perpetuity. It is made available under aCC-BY 4.0 International license.

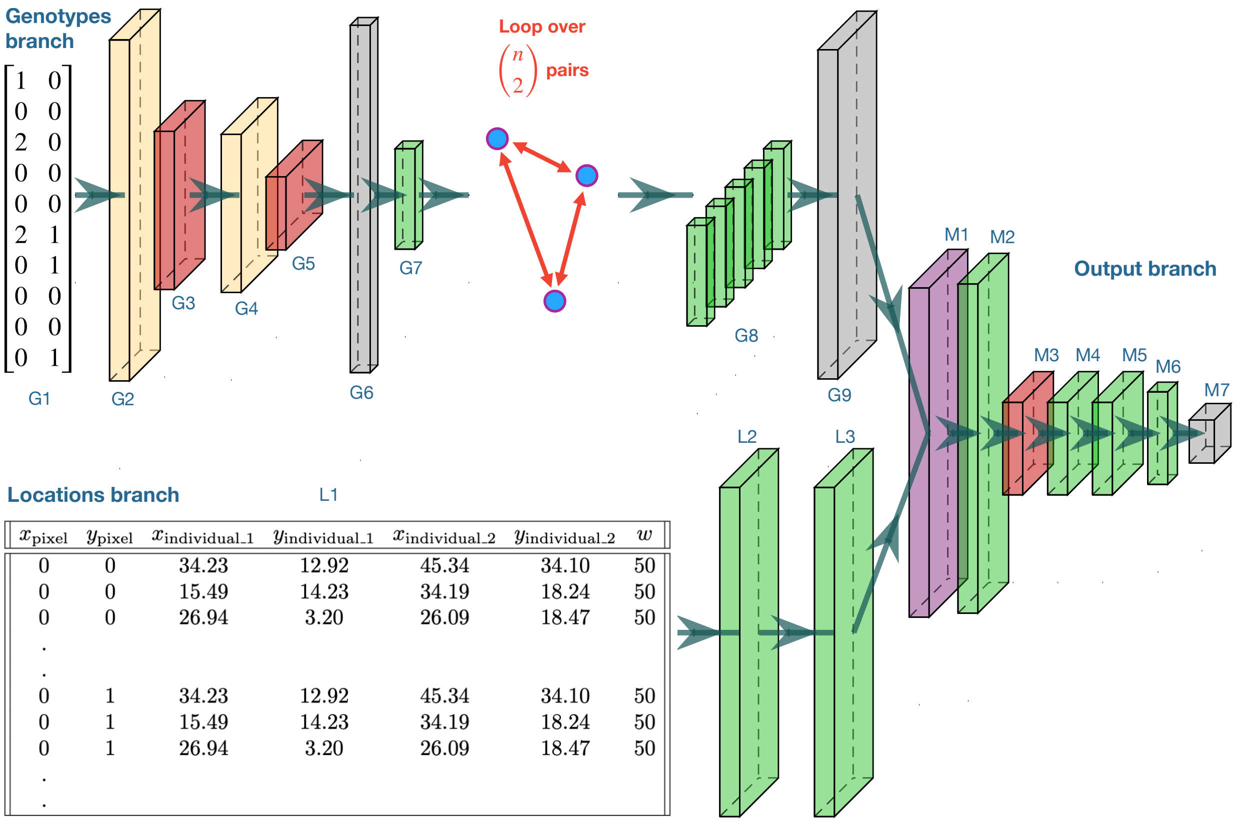

Figure S1: Extended caption for Figure 1. Visualized tensor sizes are proportional to the cube root of actual dimensions if 5,000 SNPs, 10 pairs, and map width 10. Descriptions and output sizes for each layer are described below.

- G1. (s, 2) Genotypes for a pair of individuals. This branch of the network will be repeated for multiple pairs.
- G2. (s, 64) 1D convolution, kernel size 2, 64 filters, rectified linear unit (ReLU) activation.
- G3. ($s/10$, 64) 1D average pooling, window size 10.
- G4. ($s/10$, 108) 1D convolution, kernel size 2, 64 filters, ReLU.
- G5. ($s/100$, 108) 1D average pooling, window size 10.
- G6. ($108 \times s/100$) Flatten.
- G7. (128) Dense, 128 filters, ReLU.
- G8. (128, k) Outputs from looping over pairs (only five pairs are shown).
- G9. (kw^2, 128) The outputs from k pairs are stacked together, and then duplicated for each of w^2 pixels.
- L1. (kw^2, 7) Locations table for every combination of pixel and genotype-pair (not all rows are shown).
- L2. (kw^2, 128) Dense, 128 filters, ReLU.
- L3. (kw^2, 128) Dense, 128 filters, ReLU.
- M1. (kw^2, 128) Element wise multiplication between layers G9 and L3, followed by ReLU.
- M2. (kw^2, 64) Dense, 64 filters, ReLU.
- M3. (w^2, 64) 1D pooling, every k rows.
- M4. (w^2, 64) Dense, 64 filters, ReLU.
- M5. (w^2, 64) Dense, 64 filters, ReLU.
- M6. (w^2, 2) Dense, 2 filters (linear activation).
- M7. (w, w, 2) Rearrange into a stack of two maps.

bioRxiv preprint doi: https://doi.org/10.1101/2024.03.15.585300; this version posted March 17, 2024. The copyright holder for this preprint (which was not certified by peer review) is the author/funder, who has granted bioRxiv a license to display the preprint in perpetuity. It is made available under aCC-BY 4.0 International license.

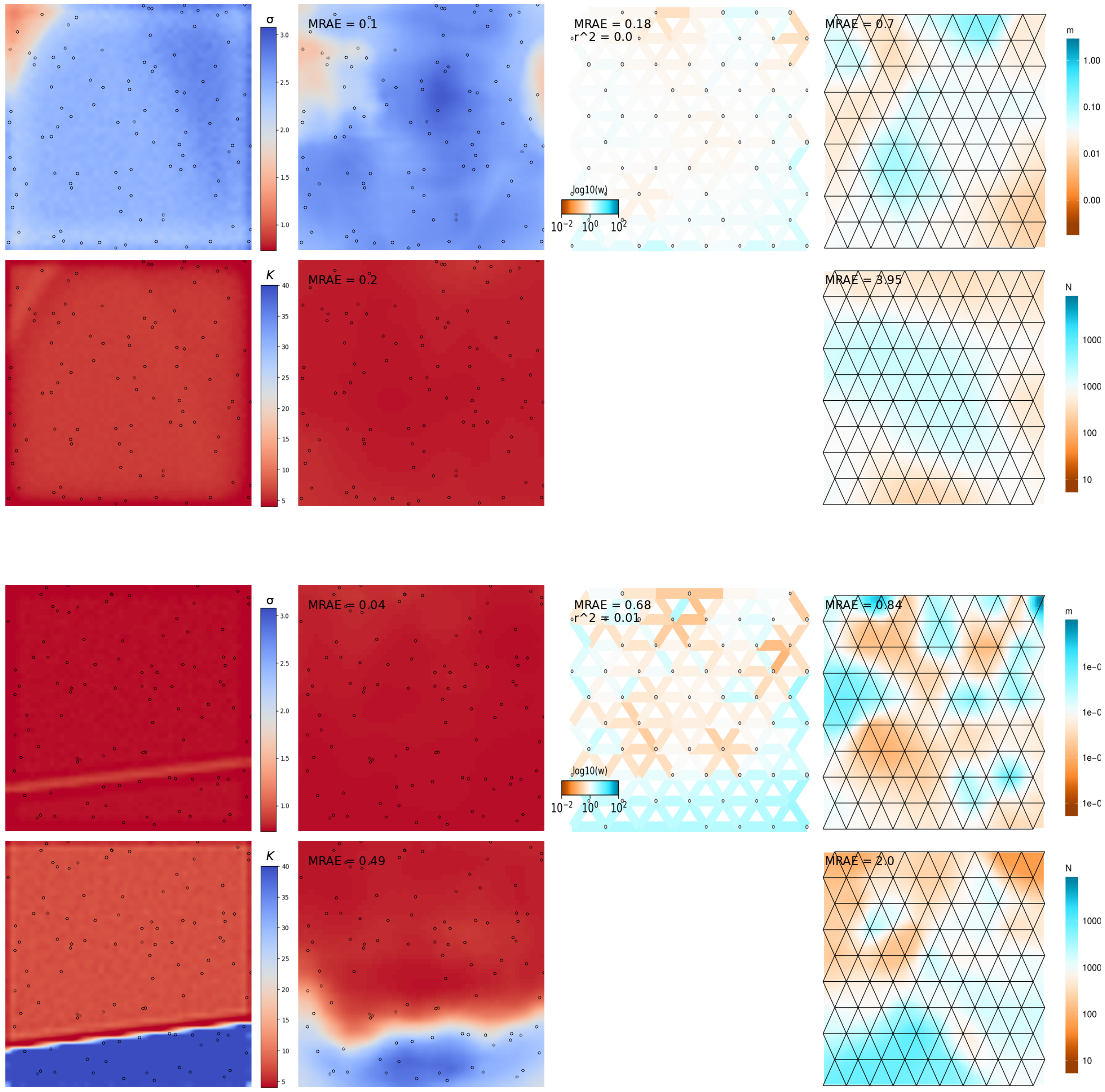

Figure S2: Predicted maps for a randomly selected, simulated test dataset. The leftmost column shows the ground truth maps for dispersal (top row) and density (bottom row). Columns 2-4 show estimated maps using three different methods: `mapNN`, `FEEMS`, and `MAPS` (respectively).

bioRxiv preprint doi: https://doi.org/10.1101/2024.03.15.585300; this version posted March 17, 2024. The copyright holder for this preprint (which was not certified by peer review) is the author/funder, who has granted bioRxiv a license to display the preprint in perpetuity. It is made available under aCC-BY 4.0 International license.

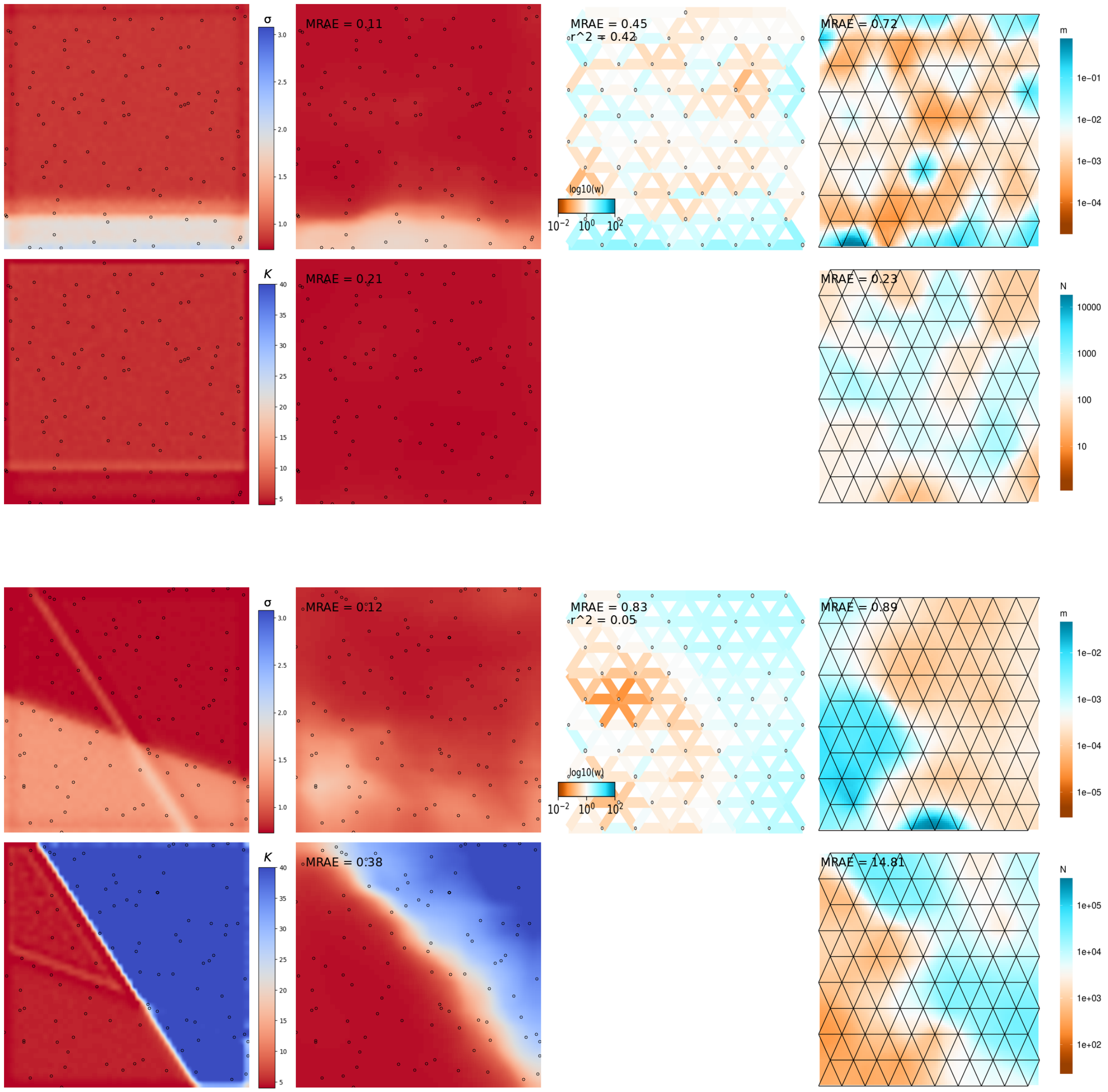

Figure S3: Predicted maps for a randomly selected, simulated test dataset. The leftmost column shows the ground truth maps for dispersal (top row) and density (bottom row). Columns 2-4 show estimated maps using three different methods: `mapNN`, `FEEMS`, and `MAPS` (respectively).

bioRxiv preprint doi: https://doi.org/10.1101/2024.03.15.585300; this version posted March 17, 2024. The copyright holder for this preprint (which was not certified by peer review) is the author/funder, who has granted bioRxiv a license to display the preprint in perpetuity. It is made available under aCC-BY 4.0 International license.

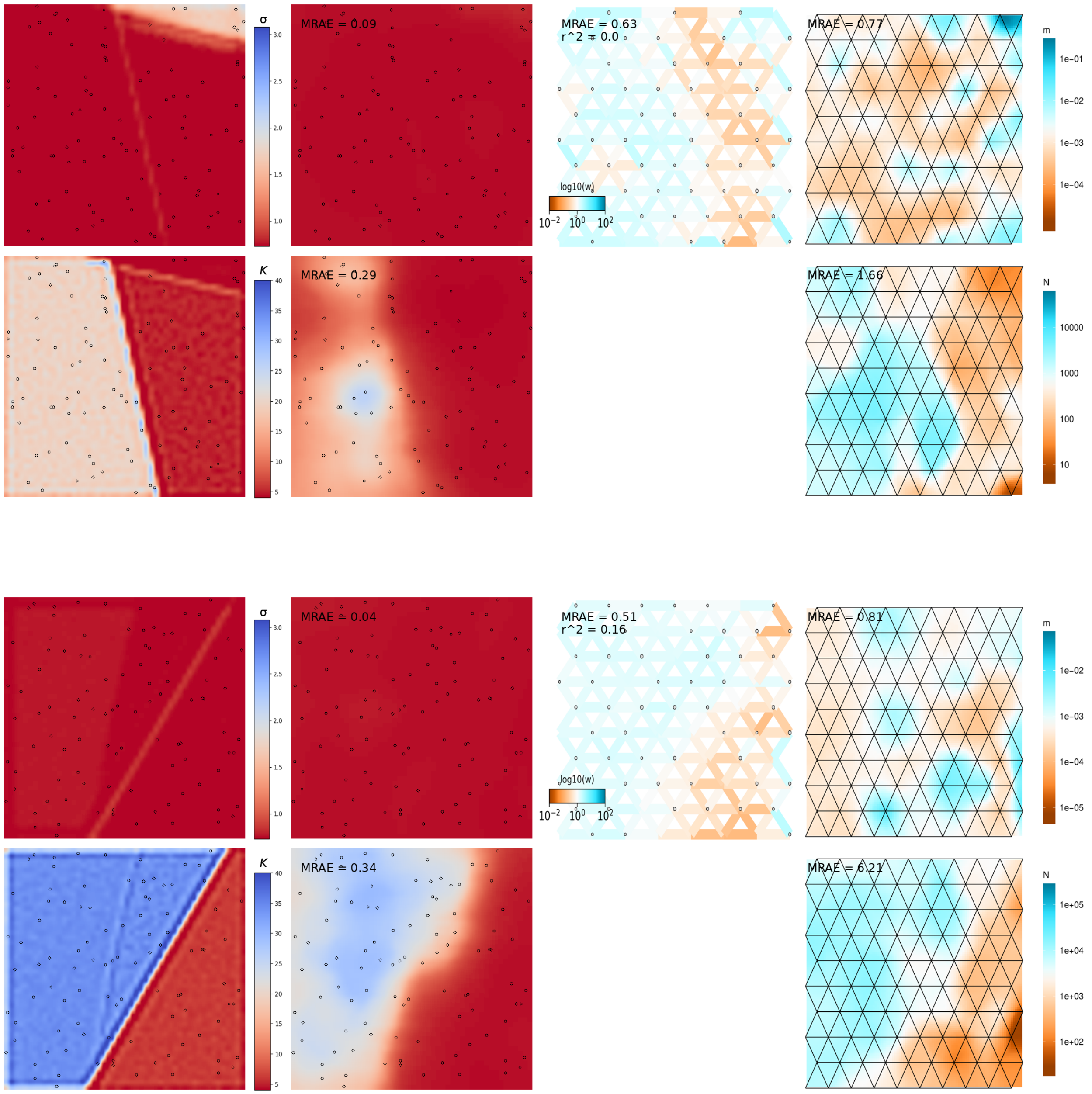

Figure S4: Predicted maps for a randomly selected, simulated test dataset. The leftmost column shows the ground truth maps for dispersal (top row) and density (bottom row). Columns 2-4 show estimated maps using three different methods: `mapNN`, `FEEMS`, and `MAPS` (respectively).

bioRxiv preprint doi: https://doi.org/10.1101/2024.03.15.585300; this version posted March 17, 2024. The copyright holder for this preprint (which was not certified by peer review) is the author/funder, who has granted bioRxiv a license to display the preprint in perpetuity. It is made available under aCC-BY 4.0 International license.

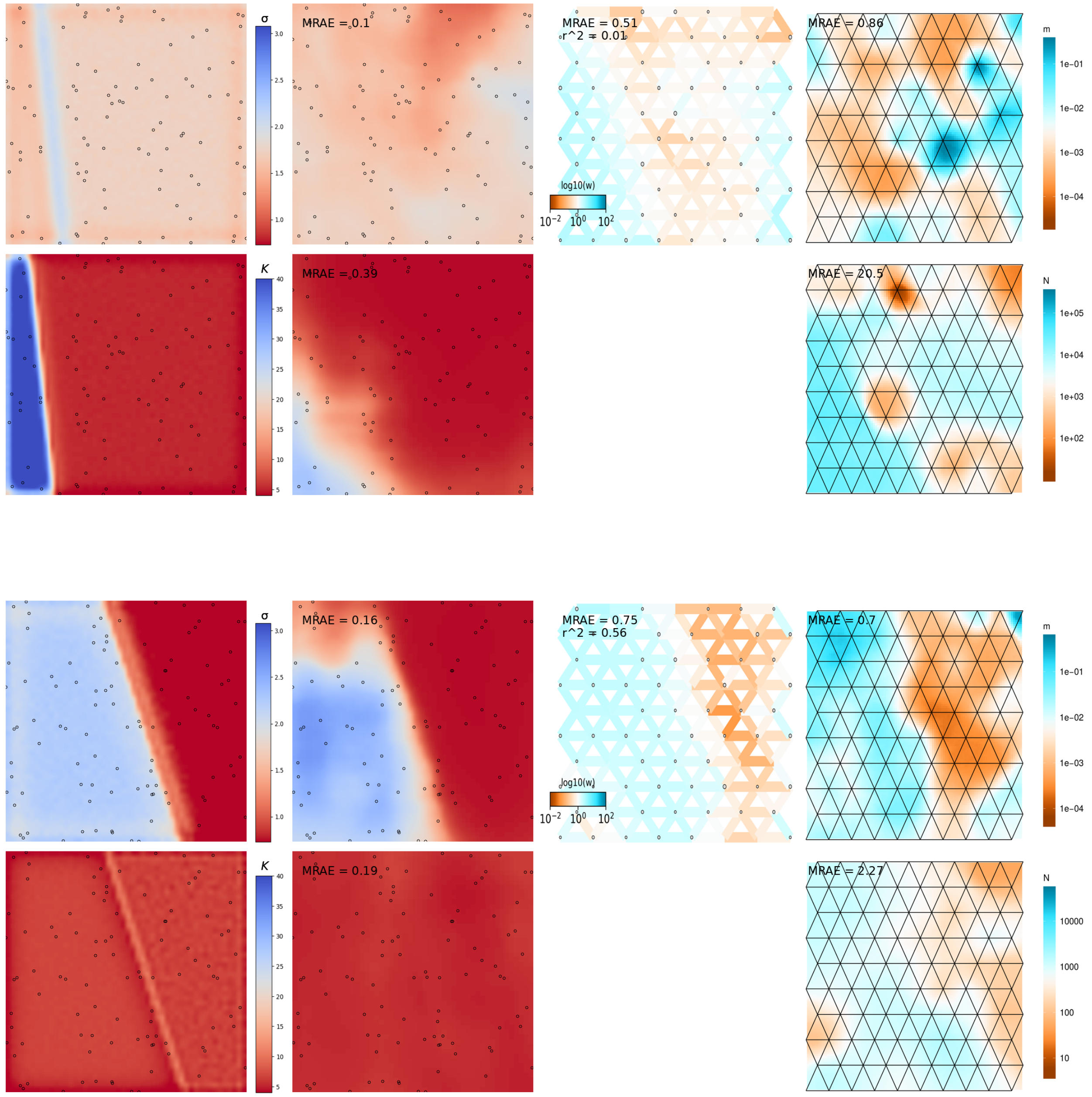

Figure S5: Predicted maps for a randomly selected, simulated test dataset. The leftmost column shows the ground truth maps for dispersal (top row) and density (bottom row). Columns 2-4 show estimated maps using three different methods: `mapNN`, `FEEMS`, and `MAPS` (respectively).

bioRxiv preprint doi: https://doi.org/10.1101/2024.03.15.585300; this version posted March 17, 2024. The copyright holder for this preprint (which was not certified by peer review) is the author/funder, who has granted bioRxiv a license to display the preprint in perpetuity. It is made available under aCC-BY 4.0 International license.

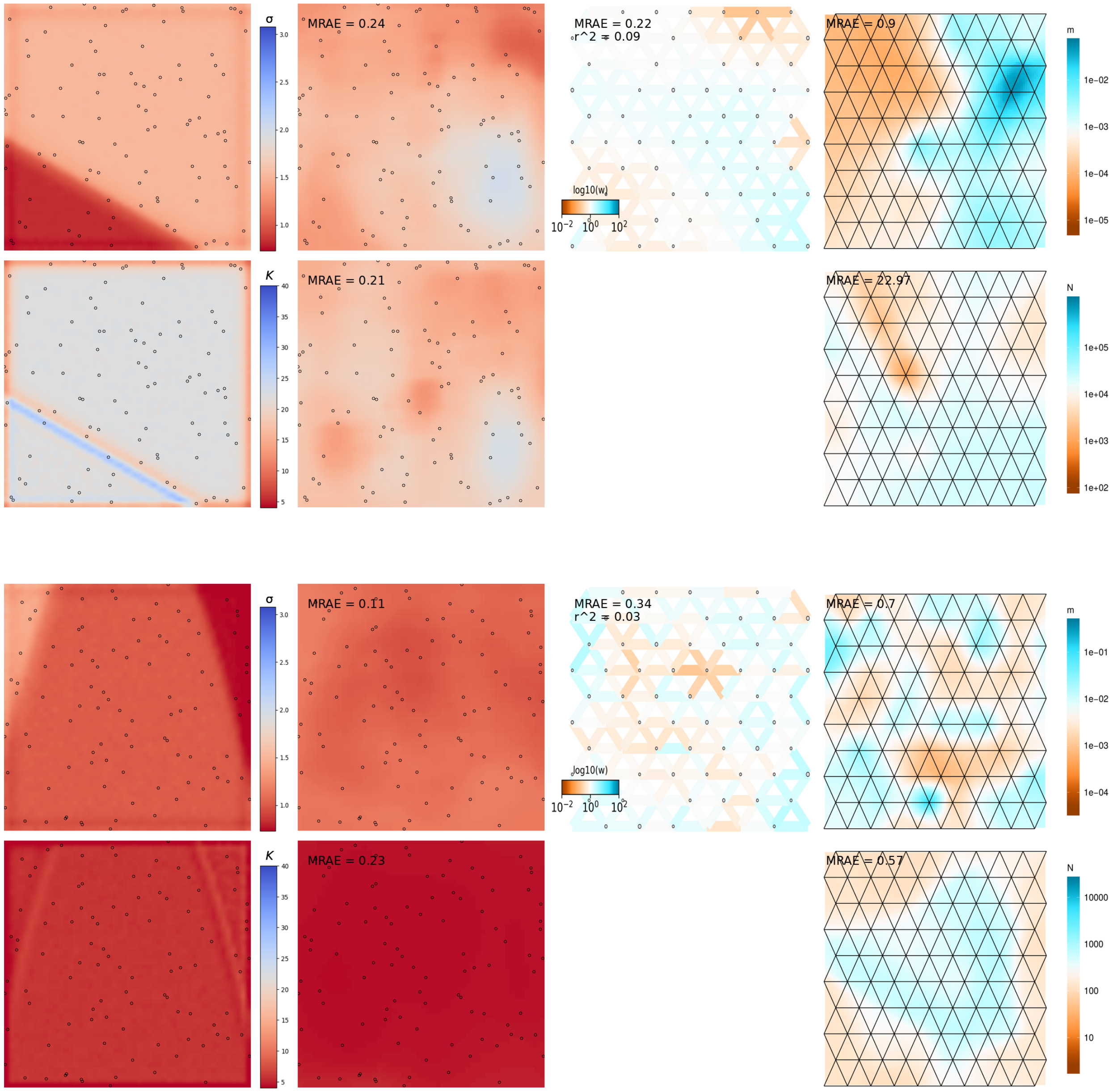

Figure S6: Predicted maps for a randomly selected, simulated test dataset. The leftmost column shows the ground truth maps for dispersal (top row) and density (bottom row). Columns 2-4 show estimated maps using three different methods: `mapNN`, `FEEMS`, and `MAPS` (respectively).

bioRxiv preprint doi: https://doi.org/10.1101/2024.03.15.585300; this version posted March 17, 2024. The copyright holder for this preprint (which was not certified by peer review) is the author/funder, who has granted bioRxiv a license to display the preprint in perpetuity. It is made available under aCC-BY 4.0 International license.

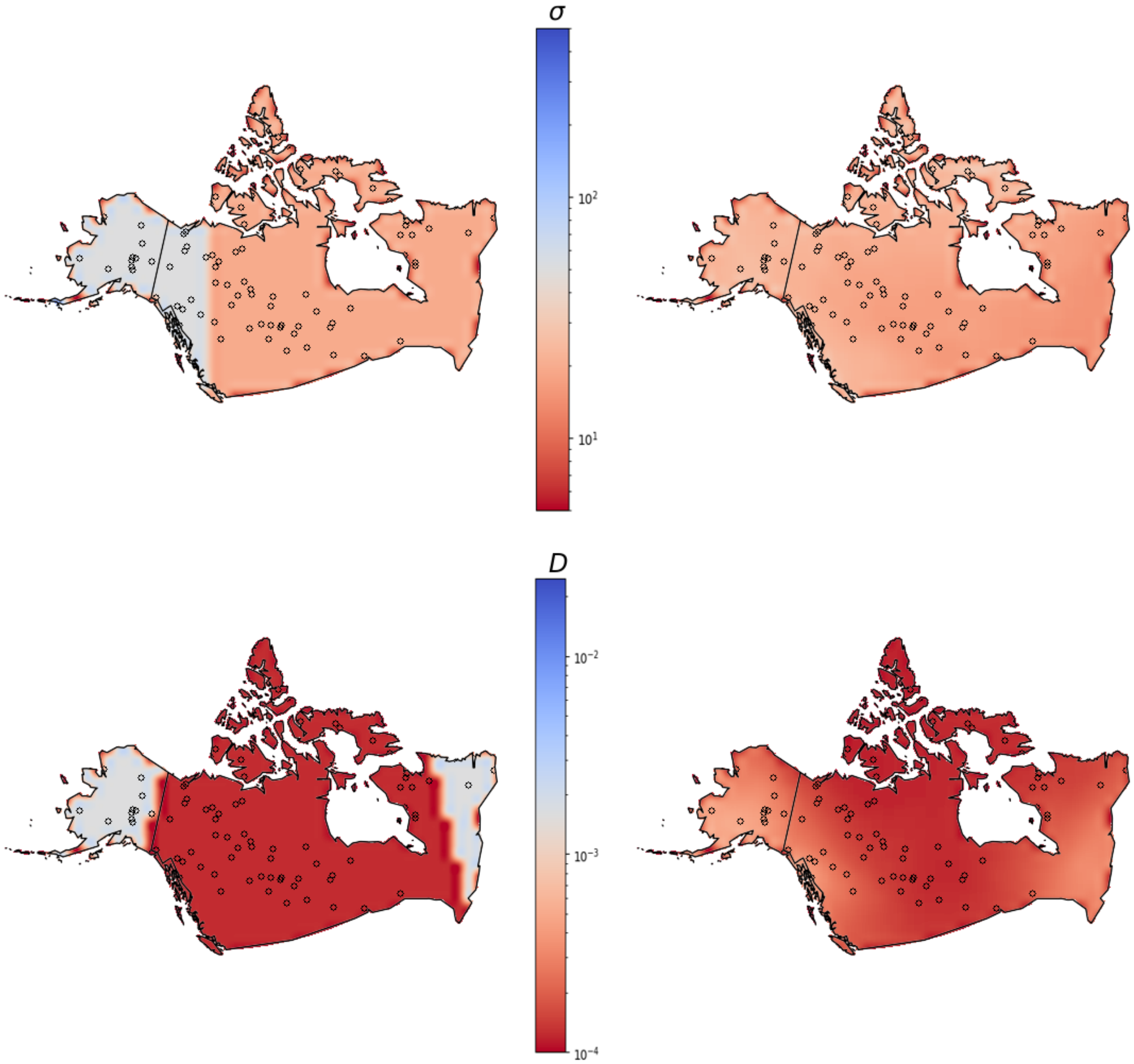

Figure S7: Predicted maps for a randomly selected, simulated test dataset for the North American grey wolf analysis. The left column shows estimated values and the right column shows uncertainty: the heat map conveys the width of each pixel-wise 95% confidence interval from parametric bootstrapping. The first row is for dispersal rate, and the second row for density.

bioRxiv preprint doi: https://doi.org/10.1101/2024.03.15.585300; this version posted March 17, 2024. The copyright holder for this preprint (which was not certified by peer review) is the author/funder, who has granted bioRxiv a license to display the preprint in perpetuity. It is made available under aCC-BY 4.0 International license.

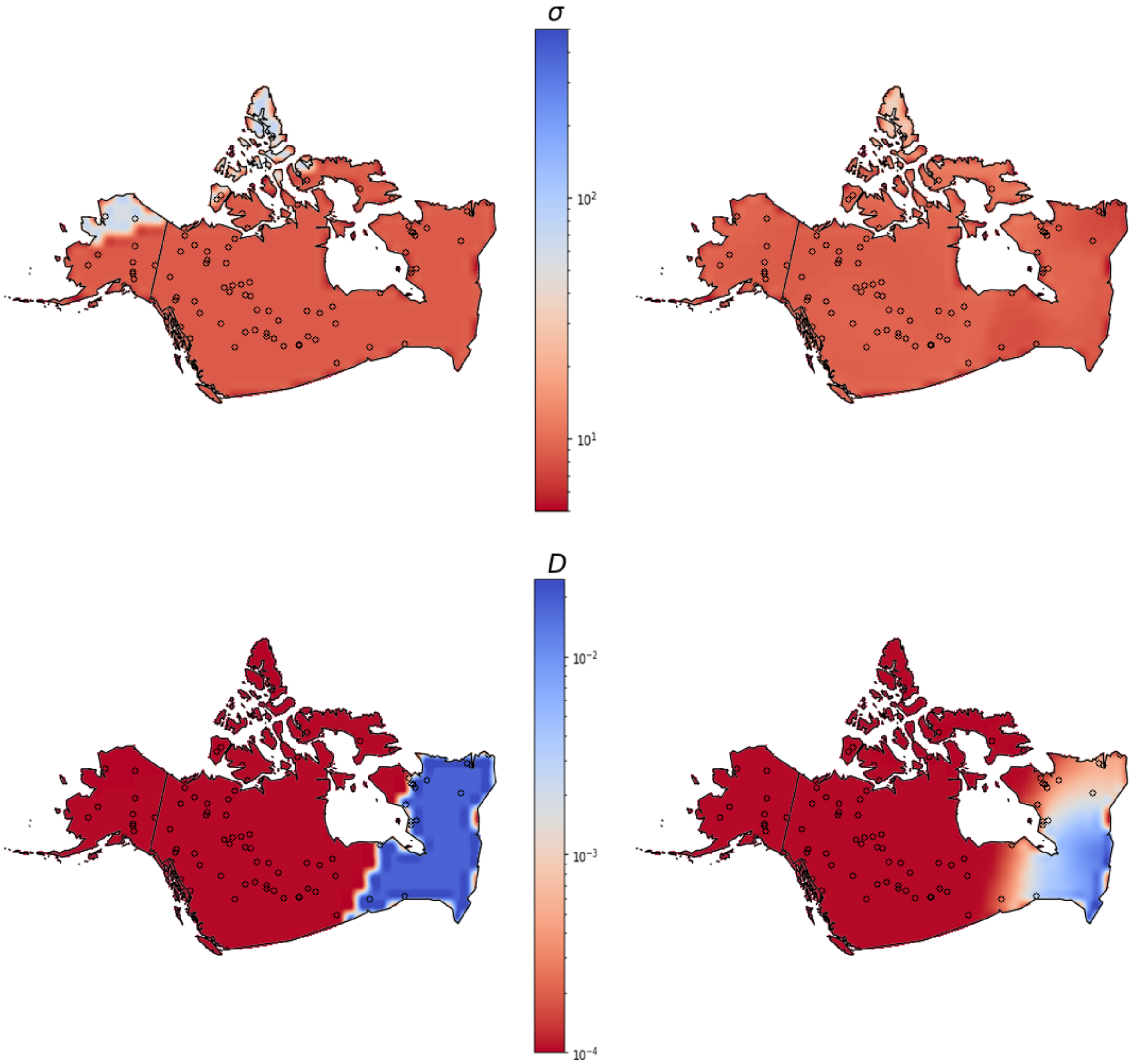

Figure S8: Predicted maps for a randomly selected, simulated test dataset for the North American grey wolf analysis. The left column shows estimated values and the right column shows uncertainty: the heat map conveys the width of each pixel-wise 95% confidence interval from parametric bootstrapping. The first row is for dispersal rate, and the second row for density.

bioRxiv preprint doi: https://doi.org/10.1101/2024.03.15.585300; this version posted March 17, 2024. The copyright holder for this preprint (which was not certified by peer review) is the author/funder, who has granted bioRxiv a license to display the preprint in perpetuity. It is made available under aCC-BY 4.0 International license.

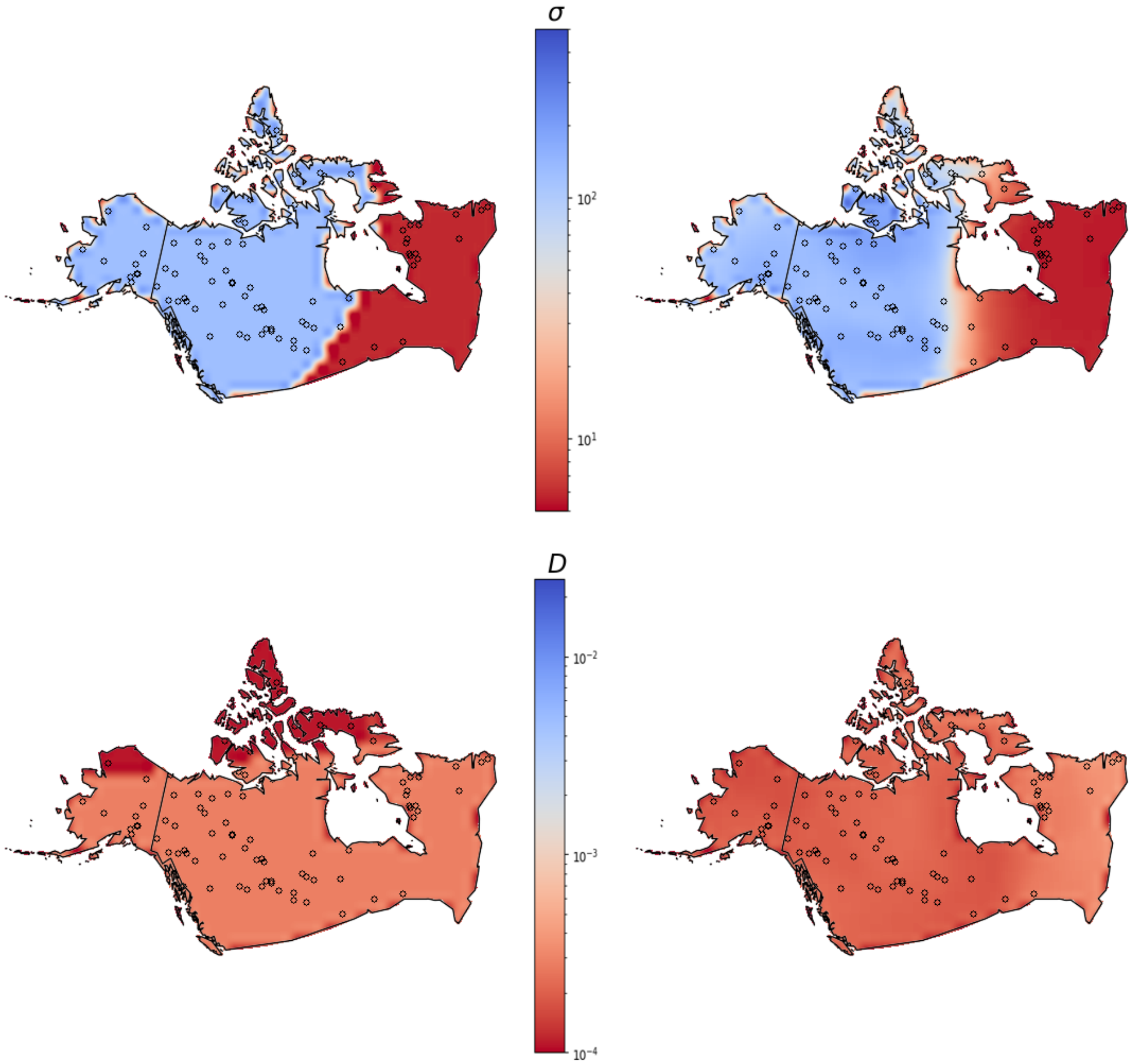

Figure S9: Predicted maps for a randomly selected, simulated test dataset for the North American grey wolf analysis. The left column shows estimated values and the right column shows uncertainty: the heat map conveys the width of each pixel-wise 95% confidence interval from parametric bootstrapping. The first row is for dispersal rate, and the second row for density.

bioRxiv preprint doi: https://doi.org/10.1101/2024.03.15.585300; this version posted March 17, 2024. The copyright holder for this preprint (which was not certified by peer review) is the author/funder, who has granted bioRxiv a license to display the preprint in perpetuity. It is made available under aCC-BY 4.0 International license.

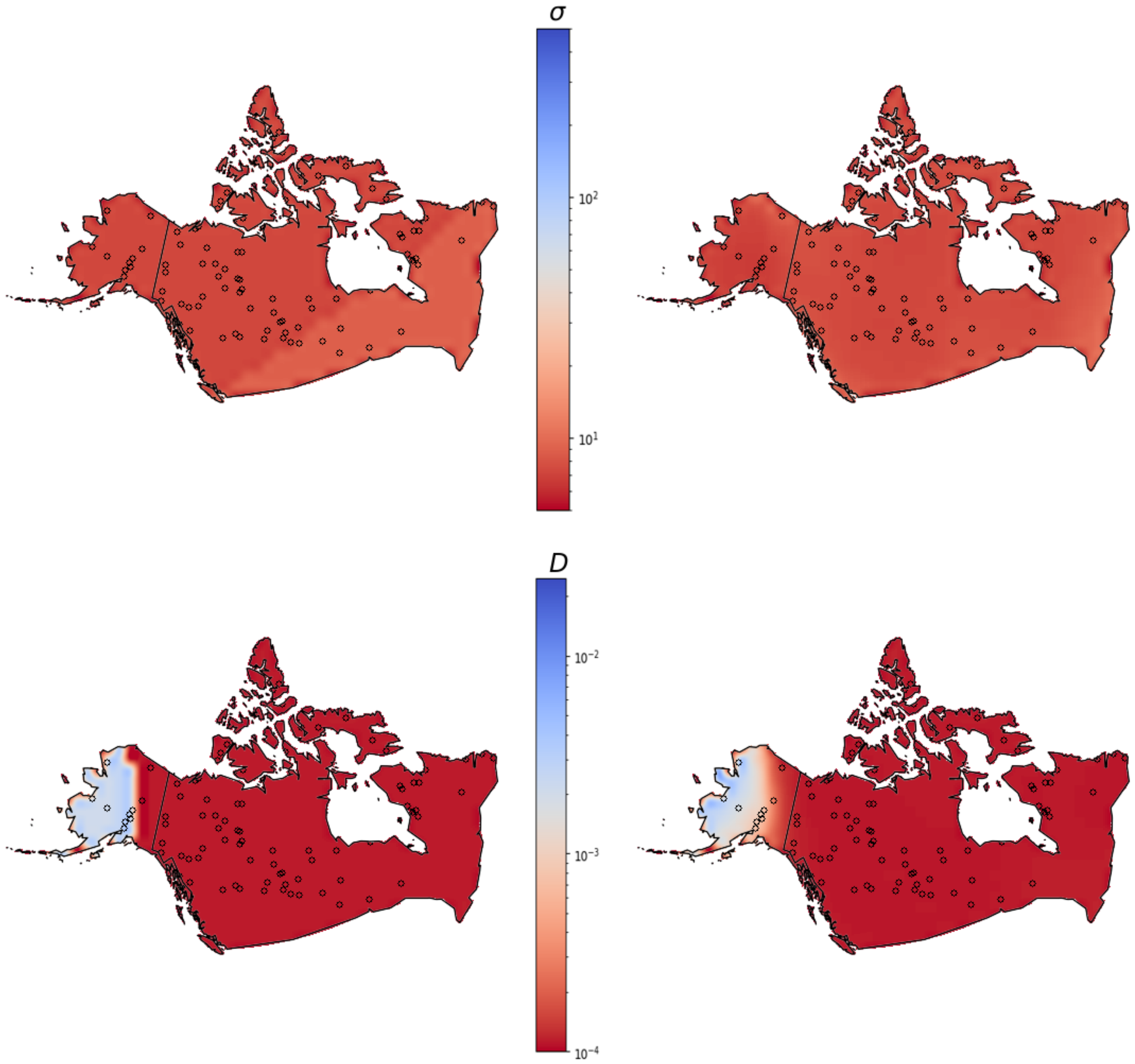

Figure S10: Predicted maps for a randomly selected, simulated test dataset for the North American grey wolf analysis. The left column shows estimated values and the right column shows uncertainty: the heat map conveys the width of each pixel-wise 95% confidence interval from parametric bootstrapping. The first row is for dispersal rate, and the second row for density.

bioRxiv preprint doi: https://doi.org/10.1101/2024.03.15.585300; this version posted March 17, 2024. The copyright holder for this preprint (which was not certified by peer review) is the author/funder, who has granted bioRxiv a license to display the preprint in perpetuity. It is made available under aCC-BY 4.0 International license.

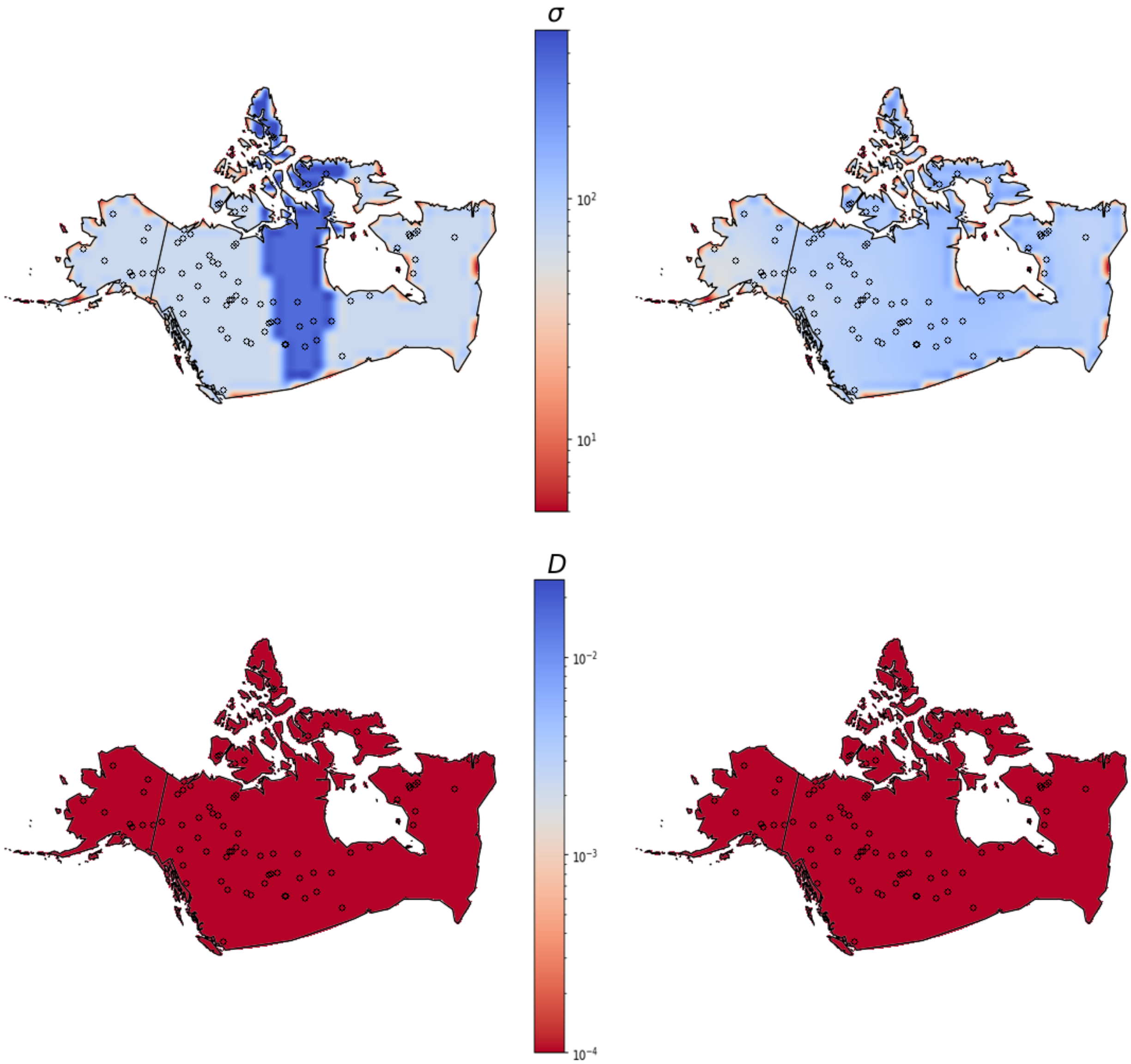

Figure S11: Predicted maps for a randomly selected, simulated test dataset for the North American grey wolf analysis. The left column shows estimated values and the right column shows uncertainty: the heat map conveys the width of each pixel-wise 95% confidence interval from parametric bootstrapping. The first row is for dispersal rate, and the second row for density.

bioRxiv preprint doi: https://doi.org/10.1101/2024.03.15.585300; this version posted March 17, 2024. The copyright holder for this preprint (which was not certified by peer review) is the author/funder, who has granted bioRxiv a license to display the preprint in perpetuity. It is made available under aCC-BY 4.0 International license.

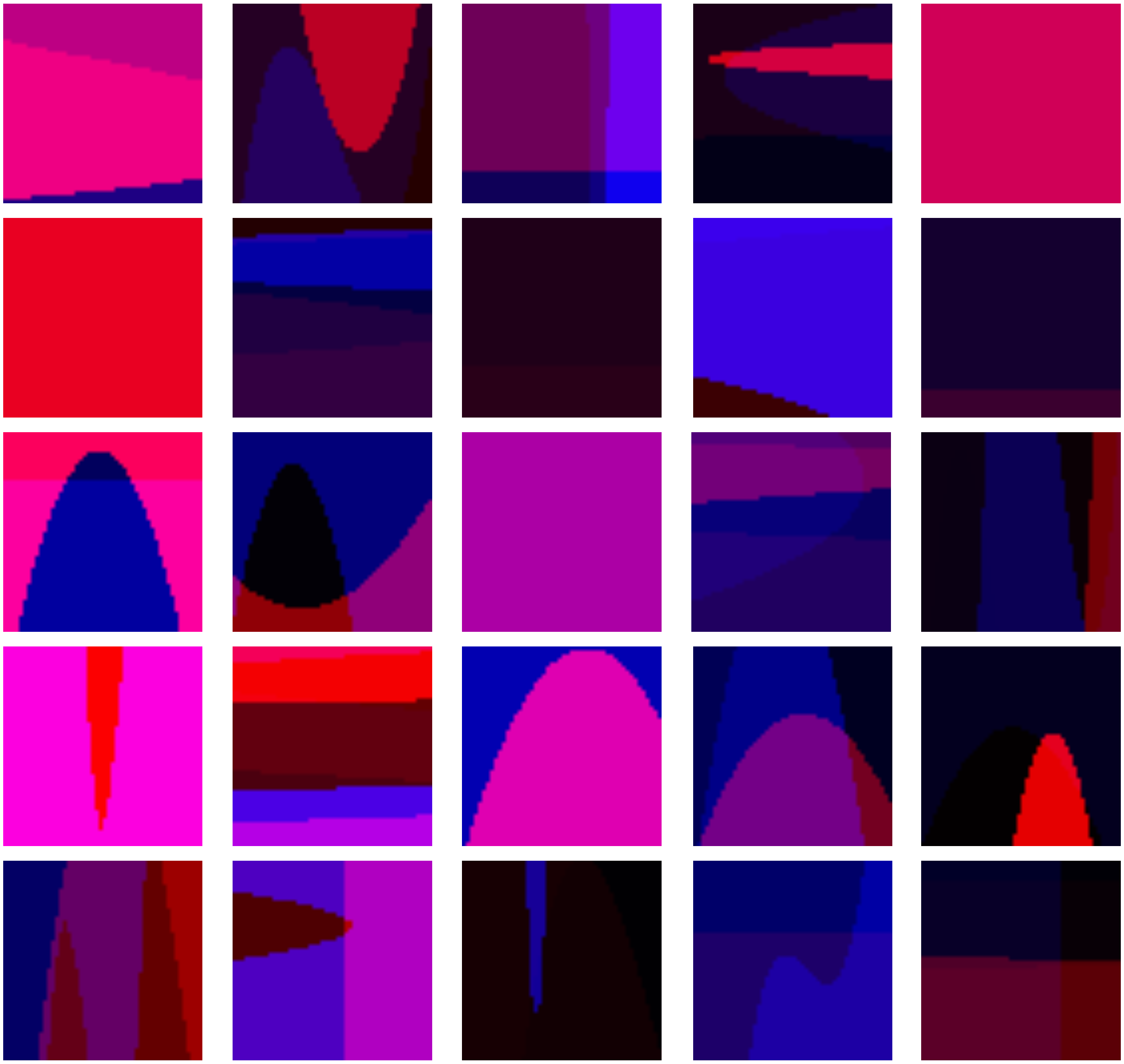

Figure S12: PNG renderings for a random selection of training maps for the benchmark analysis. The blue channel conveys dispersal rate and the red channel conveys carrying capacity.

bioRxiv preprint doi: https://doi.org/10.1101/2024.03.15.585300; this version posted March 17, 2024. The copyright holder for this preprint (which was not certified by peer review) is the author/funder, who has granted bioRxiv a license to display the preprint in perpetuity. It is made available under aCC-BY 4.0 International license.

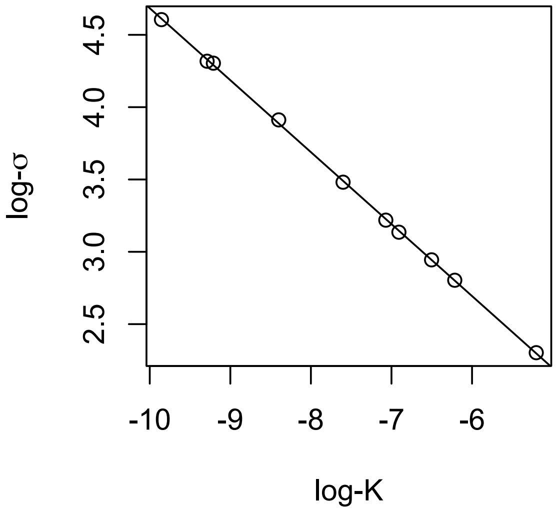

Figure S13: The minimum dispersal rate (σ) supporting a stable population for different carrying capacity (K) values.

bioRxiv preprint doi: https://doi.org/10.1101/2024.03.15.585300; this version posted March 17, 2024. The copyright holder for this preprint (which was not certified by peer review) is the author/funder, who has granted bioRxiv a license to display the preprint in perpetuity. It is made available under aCC-BY 4.0 International license.

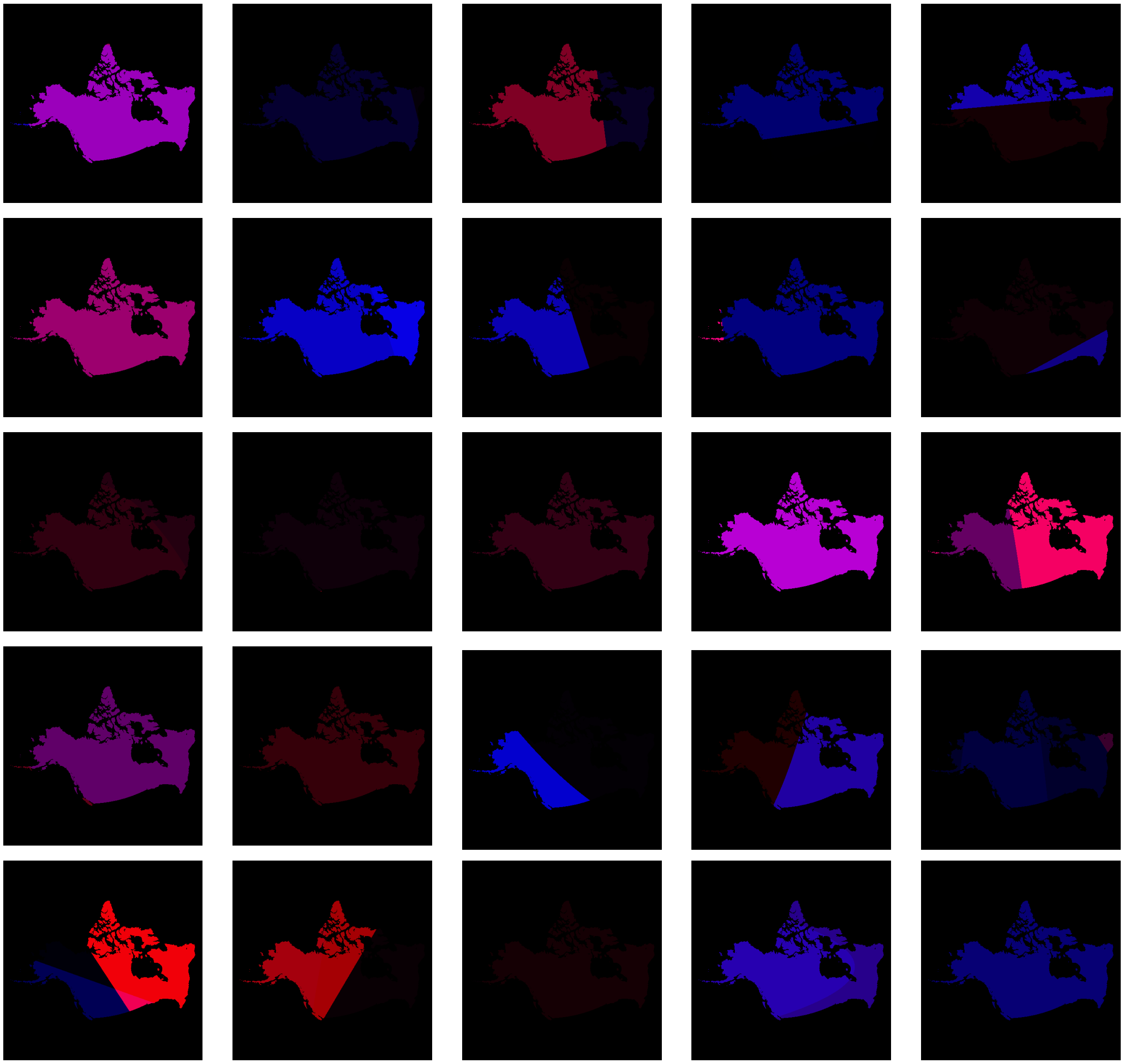

Figure S14: PNG renderings for a random selection of training maps for the North American grey wolf analysis. The blue channel conveys dispersal rate and the red channel conveys carrying capacity.
